# Tensile Properties Characterization of AlSi10Mg Parts Produced by Direct Metal Laser Sintering via Nested Effects Modeling

**DOI:** 10.3390/ma10020144

**Published:** 2017-02-08

**Authors:** Biagio Palumbo, Francesco Del Re, Massimo Martorelli, Antonio Lanzotti, Pasquale Corrado

**Affiliations:** 1 Department of Industrial Engineering, University of Naples Federico II, P.le Tecchio 80, 80125 Napoli, Italy; biagio.palumbo@unina.it (B.P.); francesco.delre@unina.it (F.D.R.); antonio.lanzotti@unina.it (A.L.); 2 MBDA Italia S.P.A., Via Calosi 105, 80070 Bacoli, Italy; pasquale.corrado@mbda.it

**Keywords:** Additive Manufacturing (AM), Direct Metal Laser Sintering (DMLS), Design of Experiments (DOE), Nested Effects Modeling (NEM), ANalysis of VAriance (ANOVA)

## Abstract

A statistical approach for the characterization of Additive Manufacturing (AM) processes is presented in this paper. Design of Experiments (DOE) and ANalysis of VAriance (ANOVA), both based on Nested Effects Modeling (NEM) technique, are adopted to assess the effect of different laser exposure strategies on physical and mechanical properties of AlSi10Mg parts produced by Direct Metal Laser Sintering (DMLS). Due to the wide industrial interest in AM technologies in many different fields, it is extremely important to ensure high parts performances and productivity. For this aim, the present paper focuses on the evaluation of tensile properties of specimens built with different laser exposure strategies. Two optimal laser parameters settings, in terms of both process quality (part performances) and productivity (part build rate), are identified.

## 1. Introduction

Additive Manufacturing (AM) is considered as the opposite of subtractive manufacturing: the final functional part is gradually built by adding material layer upon layer, instead of removing it.

AM processes offer several technical and economic benefits compared to traditional subtractive manufacturing processes. They have the capability to produce complex shapes which are not feasible with traditional manufacturing processes. The geometric freedom associated with AM provides new possibilities for the part design. AM processes, integrated with techniques for topology optimization and other design methods can generate complex shapes, and potentially allow saving time, material and costs.

Direct Metal Laser Sintering (DMLS), a typical Powder Bed Fusion (PBF) process based on the layer-by-layer spreading and subsequent laser sintering of metal powders [1], was considered in this paper.

By laying down material, usually in subsequent layers from the bottom up, it is possible to effectively “grow” nearly-finished functional parts, rather than machining material away or using dies and molds, directly from a 3D-CAD model.

**Figure 1:**
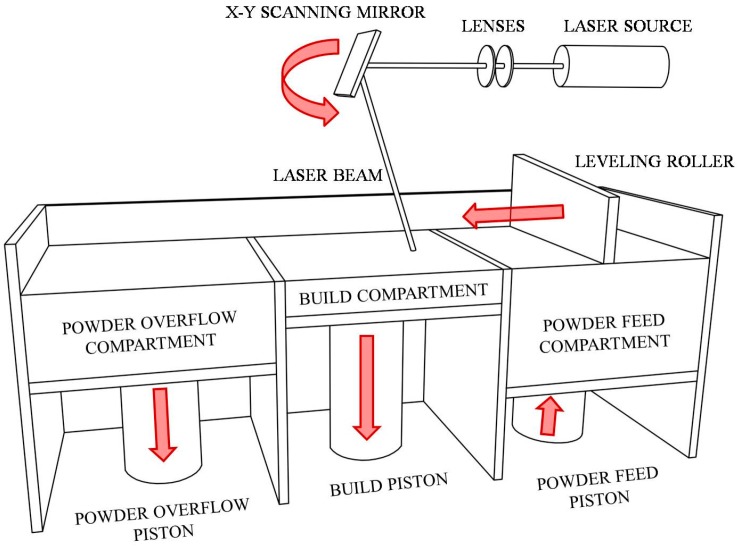
Functional scheme of Direct Metal Laser Sintering process.

Each powder layer is subsequently spread by a roller, or coater, and sintered by means of a laser beam, based on information contained in the CAD file, until the desired three-dimensional shape is achieved. A manageable functional scheme of the DMLS process is shown in Figure 1.

Many papers available in the literature about laser sintering/melting of Aluminum powders show an unceasing effort in testing effects of different topological [2,3,4,5,6] and processing parameters on the produced parts features. In particular, the experimental activities concerning effects of different laser exposure parameters, like those proposed in this paper, deal with the identification of the most suitable amount of energy to be delivered to the powder bed for a proper melting.

Note that the maximum power of the used laser source affects the choice of laser exposure parameters in terms of achievable energy densities and build rates.

Most of the papers concern PBF equipment with laser powers of 200–250 W. In [2], two different strategies were used for skin and core exposures in order to produce dense AlSiMg parts, with energy densities, ψ_d_, of 44.44 J/mm^3^ and 47.79 J/mm^3^, respectively. A similar investigation was carried out in [7], where the same laser parameters, therefore the same energy density, were used for the core exposure, but different exposure strategies were tested for the up-skin exposure, with ψ_d_ ranging from 16 J/mm^3^ to 79.17 J/mm^3^. In [3], an optimal density range was found between 50 J/mm^3^ and 65 J/mm^3^, with a lack of consolidation for lower values and keyhole formations for higher ones. In [8], effects of different part build angles and heat treatments on AlSi10Mg parts properties were investigated, using an optimized set of processing parameters corresponding to 66.67 J/mm^3^. Similarly, in [4,9], the optimal energy density used was 45.35 J/mm^3^. In [5,10], the range 48–70 J/mm^3^ was identified as the threshold which separates partial and full melting conditions for different additively manufactured aluminum alloys. This optimized process window was later narrowed in [6,11] and again 66.67 J/mm^3^ was identified as the most suitable energy density value for the best material consolidation.

In [12,13], effects of laser processing parameters on the achievable densification of aluminum alloys were studied to obtain nearly full dense parts by using laser sources of only 50–100 W providing the powder bed with energy densities of ≈37 J/mm^3^ (at 50 W) and 45–78 J/mm^3^ (at 100 W), depending on the selected alloy.

In case of laser sources with power up to 250 W, it was not possible to achieve build rates higher than 4.5 mm^3^/s: they generally resulted in the range of 2–4.4 mm^3^/s.

Conversely, high power (up to 1 kW) Selective Laser Melting of aluminum parts was investigated in [14], showing the possibility to achieve material densities higher than 99.5% with ψ_d_ = 42.35 J/mm^3^ and extremely high build rates (up to 21 mm^3^/s).

Literature review has returned no information on PBF processes on aluminum alloys carried out with a 400 W source, like the one used in this research, but the possibility of using moderately higher power is expected to deliver equally performing materials with higher productivity.

Conventional trial-and-error experimental approaches are not very suitable: high costs of both raw materials and equipment, the lack of knowledge among practitioners, the need to test many processing parameters and the long time required for the execution of experimental campaigns are just some technological constraints which make the characterization and optimization of Additive Manufacturing processes very expensive.

Previous investigations which have been carried out by the authors, as well as several others which have been found in literature, have underlined benefits of a statistical approach for the characterization of AM processes. In [15,16,17], ANalysis Of VAriance (ANOVA) and Response Surface Method (RSM) were used to evaluate the influence of Fused Deposition Modeling (FDM) process parameters on the achievable features of polymeric parts and to identify the optimal process window. Similar techniques were implemented in [3,18] on PBF processes for metals in order to optimize mechanical and surface finishing properties of aluminum parts.

This paper focuses on the definition and implementation of statistical methods to study the effects of different laser exposure strategies on the achievable tensile properties of AlSi10Mg parts produced by the DMLS technology, as well as on the process productivity itself, since the research activity was developed within a corporate context. The experimental campaign was planned on the basis of Design of Experiments (DOE) concepts, while Nested ANalysis of VAriance (Nested ANOVA) technique was used to reliably assess the effects of selected control factors.

## 2. Materials and Methods

The experimental study was carried out on an Al-based alloy, the EOS Aluminum AlSi10Mg, provided by the EOS GmbH Electro Optical Systems in the form of a gas-atomized metal powder.

The EOS AlSi10Mg is a typical casting alloy, which is ideal for light parts with thin walls, complex geometries and specific thermal properties. The chemical composition of the experimental material, according to the datasheet provided by the producer, is shown in Table 1.

EOS provides, for each alloy combined with specified equipment, an optimized set of processing parameters, which are called Part Property Profile (PPP) and a Stress Relieving Cycle (SRC) procedure. PPPs are commonly masked and locked, which means that parameters settings are not visible, nor editable. Table 2 summarizes PPP and SRC available for EOS AlSi10Mg.

All specimens were produced by using the EOSINT M280 equipment for the DMLS process, provided by the EOS GmbH, together with the entire secondary support instrumentation.

The main characteristics of the equipment are listed below:

- Laser Source: Yb fiber laser 400 W
- Beam wavelength: 1060–1100 nm
- Beam spot size: ≈100 μm
- Scanning exposure area: 250 mm × 250 mm
- Scan speed: up to 7000 mm/s
- Z-axis stroke: 325 mm (including the platform)

In order to achieve minimum certified and repeatable mechanical performances and minimize any deviation from expected values, all produced specimens were post-processed using the SRC procedure suggested by EOS, as well as 100% of new powder.

### 2.1. Pre-Design Phase and Pre-Design Guide Sheets

Practitioners’ experience on the DMLS process has greatly improved over the years but it still reveals poor knowledge about laser exposure parameters (such as laser powers, scan speeds, scan distances, etc.) which are hard to be precisely known and controlled within the production environment.

In such cases, according to the authors, an accurate pre-design is the keystone to maximize achievable experimental results, since it allows all available expertise to be systemized, by means of well-defined operative and analysis procedures, thus reducing efforts, costs and time for this kind of process characterizations [19,20].

On the basis of guidelines proposed by Coleman and Montgomery in [21], pre-design guide sheets were used during the whole investigation, from the design to the execution of the experiment. All experimenters filled in and constantly updated these pre-design guide sheets in order to integrate statistical and technological skills so that to cope with both theoretical and technical constraints.

### 2.2. Selection of Response Variables

In order to assess effects of different laser exposure strategies on the achievable part features, experimenters decided to focus their attention on the following response variables: Density, D; Yield Strength, R_p_ 0.2%; Ultimate Tensile Strength (UTS); and Elongation at Break, A.

Density measurements were carried out by means of the Archimedes’ principle on specimens produced for tensile tests, in compliance with the ISO 3369:2006 standard [22].

**Figure 2:**
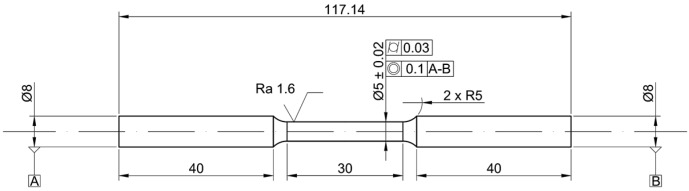
Tensile test specimen according to ISO 6892-1:2016 and ASTM E8/E8M-16a.

Specimen geometry (see Figure 2) and testing method for tensile test were selected according to literature review [23,24,25], as well as to comply to ISO 6892-1:2016 [26] and ASTM E8/E8M-16a [27] standards. Instron 1185 testing machine was used to evaluate tensile properties at room temperature, with a displacement rate of cross head of 0.0075 mm/s.

### 2.3. Choice of Control Factors and Experimental Plan

The number of process parameters related to PBF processes is so high that their characterization within a single experimental activity is almost impossible. Just to give an example, the most effective ones concern raw materials characteristics, topological aspects and interplay between the laser beam and metal powders. Since performing a comprehensive investigation on all the characterization parameters involved in this kind of processes is not the goal of this research, the attention was focused, among the large number of editable laser parameters, on settings for stripes exposure, namely: laser power, P_L_; layer thickness, L_t_; hatch distance or spacing, H_d_; and scan speed, v_s_.

Generally, when designing an experiment, it is possible to associate each control factor with an appropriate range in which to evaluate the performance of selected response variables: the resulting experimental region is equivalent to the product of such regular intervals and it is defined *regular* when it appears as a cube, or a parallelepiped, in *n* dimensions (where *n* is the number of factors of the experimental plan).

Conversely, when considering the laser sintering process, the aforementioned control factors are strictly connected with respect to the minimum energy density, ψ_d_ (measured in J/mm^3^), to be delivered to the powder bed for a proper sintering, as showed in the following Equation (1):

(1) $$\psi_{d}=\frac{P_{L}}{L_{t}\cdot H_{d}\cdot v_{s}}$$

Experimental factors are defined as related to one another when the interval of analysis of some of these factors depends on the level (or levels range) of other factors. This implies the presence of an irregular experimental region in which the analysis of the main effects and interactions is influenced by this peculiarity [28,29].

In such cases, the implementation of a conventional full factorial plan would imply the use of narrow intervals in which to vary controlling factors, or the use of larger intervals, resulting in several useless runs (out of the experimental zone).

The Nested Effects Modeling (NEM) approach allows this problem to be solved, by selecting levels of a factor (slid factor) as a function of the levels selected for another one (related factor). This approach has the advantage of expanding the experimental region and consequently it becomes more reliable to appreciate effects of control factors on the output variable. Moreover, it is possible to avoid collinearity issues when estimating effects of related factors, by using Nested ANOVA instead of conventional methodologies.

**Figure 3:**
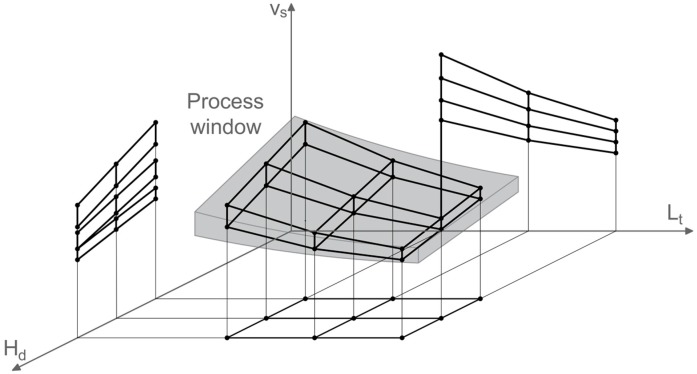
Irregular experimental region of designs with nested effects.

Since it was not possible to set laser power levels higher than the default one (testing lower values was not useful for the investigation), experimenters decided to opt for a Nested Design, by nesting levels of the scan speed within those selected for the hatch distance and the layer thickness. A graphical representation of the implemented nested design is shown in Figure 3.

The use of the NEM approach allowed experimenters to keep all defined treatments within the energy density range of 47–65 J/mm^3^, which has been identified by literature reviews as the most suitable for a proper sintering with high build rates, given the available experimental equipment. In addition, this range was found to contain the energy density value corresponding to the default exposure profile (EOS AlSi10Mg Speed 30 μm) provided with AlSi10Mg, which should already ensure high productivity.

Table 3 shows the implemented experimental plan (in coded units for company confidentiality). For each treatment, the corresponding energy density and the Estimated Build Rate (EBR) were reported.

EBRs (measured in mm^3^/s) were calculated by the following Equation (2):

(2) $$\mathrm{EBR}=L_{t}\cdot H_{d}\cdot v_{s}\;$$

Even if ψ_d_ and EBR are proportional to each other, they may be useful to provide valuable information depending on different needs. The first one indicates the technological constraint to avoid bad zones of the experimental region, while the second can be seen as a productivity index, which is crucial in an industrial environment, such as the one where this research activity was developed.

Treatment IV in Table 3 corresponds to the EOS AlSi10Mg Speed 30 μm Part Property Profile, which was considered as the reference treatment to compare experimental results.

### 2.4. Statistical Technique for Data Analysis

As previously said, the Nested ANOVA allows the effect of the nested factor for each level combination of its related factors to be modeled, avoiding collinearity issues when accounting for the relationship among related factors. On the other hand, since each level of the slid factors is not present for each level of corresponding related factors, second order interactions cannot be estimated.

The linear statistical model for the implemented nested design is the following [30]:

(3) $$y_{\mathrm{ijk}}=\mu +\tau_{i}+\beta_{j\left(i\right)}+\gamma_{k\left(\mathrm{ij}\right)}+\varepsilon_{l\left(\mathrm{ijk}\right)}(i=1,\;\ldots ,\;a\;;\;j=1,\;\ldots ,\;b\;;\;k=1,\;\ldots ,\;c\;;\;l=1,\;\ldots ,\;n)$$

where

- μ is the global mean value;
- τ_i_ is the effect of the i-th level of L_t_;
- β_j(i)_ is the effect of the j-th level of H_d_ within the i-th level of L_t_;
- γ_k(ij)_ is the effect of the k-th level of v_s_ within the j-th level of H_d_ and the i-th level of L_t_; and
- ε_l(ijk)_ is the aleatory variable experimental error.

Table 4 shows the corresponding Nested ANOVA table applied to the implemented experimental design.

Variance Inflation Factors (VIFs) and determination coefficients R^2^ and Adjusted R^2^) were used to check the robustness of all obtained results. VIFs give information about the correlation among model predictors, therefore of how much regression analysis is affected by the collinearity. Predictors are not correlated when VIF = 1, moderately correlated when 1 < VIF < 5 and highly correlated when VIF > 5. When VIF is higher than 10, regression results are excessively affected by the collinearity. Determination coefficients are used in regression analysis to indicate how well the model explains the observed variability: the higher the coefficient, the higher the percentage of variability which is explained by the model. Furthermore, it was possible to assess the contribution of each term included in the regression model to the output variance by means of variance-based Sensitivity Indices (SI), which measure the effect of the variation of just on term while averaging over variations in other input variables. In order to obtain a fractional contribution, the variance of each single term is standardized by the total variance observed for the selected response variable [31].

### 2.5. Executed Experimental Campaign

Since it was not possible to produce specimens with different layer thicknesses within the same run, three test jobs were produced, each one hosting three replicates of six treatments characterized by the same thickness, for a total of 18 tensile bars with +1 mm diameter to be tested after machining. 

**Figure 4:**
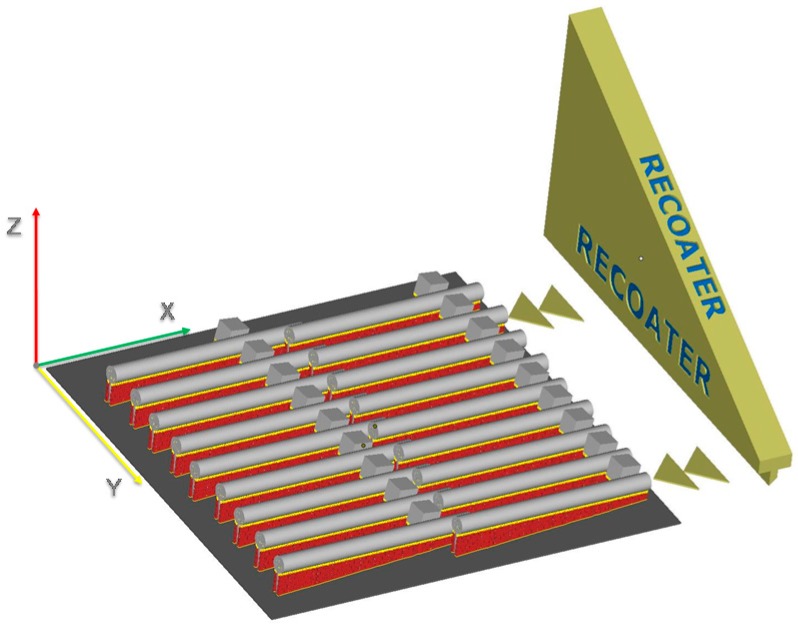
Printing job layout.

The position of specimens referring to a particular treatment was completely randomized within each printing job, in order to eliminate the influence of their position on the printing plate. In addition, all specimens were produced with a build angle of 4°, in order to minimize residual stresses, and with an angle of 4.78° with respect to the re-coating direction, to minimize fluttering effects due to the coater motion. The laser travel direction was rotated by 67° each layer. The printing job layout is shown in Figure 4.

## 3. Results and Discussion

### 3.1. Statistical Results

ANOVA on collected data was carried out at a confidence level of 95% (α = 0.05) by using the Minitab^®^ 17 statistical software, after outliers (unusual observations) removal and diagnostic checking of residuals, which appeared to be randomly scattered at about zero and compliant with the normality assumption.

The difference between a sample statistic and a hypothesized parameter value is statistically significant if the performed hypothesis test suggests this difference is too unlikely to have occurred by chance. After performing the ANOVA, the statistical significance of an effect can be assessed by looking at the test’s *p*-value, which is the probability of obtaining a test statistic at least as extreme as the one which was actually calculated from the sample, if the null hypothesis is true. If the *p*-value is below the specified significance level (α = 0.05), the difference is declared to be statistically significant and the test’s null hypothesis is rejected [30,32]. The more the *p*-value approaches zero the more the corresponding effect results significant.

Nested ANOVA results for the analyzed response variables were reported in Table 5, Table 6, Table 7 and Table 8. VIFs and SIs were reported in the last two columns, while determination coefficients in the last row.

As expected, all calculated VIFs resulted close to 1, which means that the estimation of effects is not affected by the collinearity. Moreover, determination coefficients for Yield Strength and Ultimate Tensile Strength were particularly high, therefore the corresponding linear regression models result reliable in explaining the observed variability. Conversely, they resulted relatively low for Density and Elongation at Break. SIs pointed out the predominant effect of layer thickness on the variability of analyzed material properties, except for the material density which resulted more affected by the hatch distance. In addition, high SI values of the experimental error for Density and Elongation at Break confirmed the presence of a residual variability which is not well explained by the regression model.

**Figure 5:**
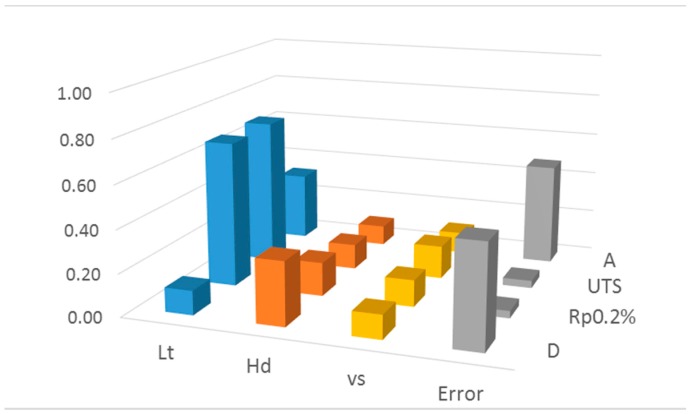
Histogram of ANOVA terms contribution on observed variability.

Table 9 summarizes significant effects (α = 0.05) for the analyzed material characteristics. Effects which resulted statistically significant or negligible were marked as “✔” and “✖”, respectively. Moreover, Figure 5 shows the contribution of each term given by Sensitivity Indices. 

All numerical data in the following plots were masked for company confidentiality. Only the reference scale was reported, for each response variable, to appreciate the range of variations.

**Figure 6:**
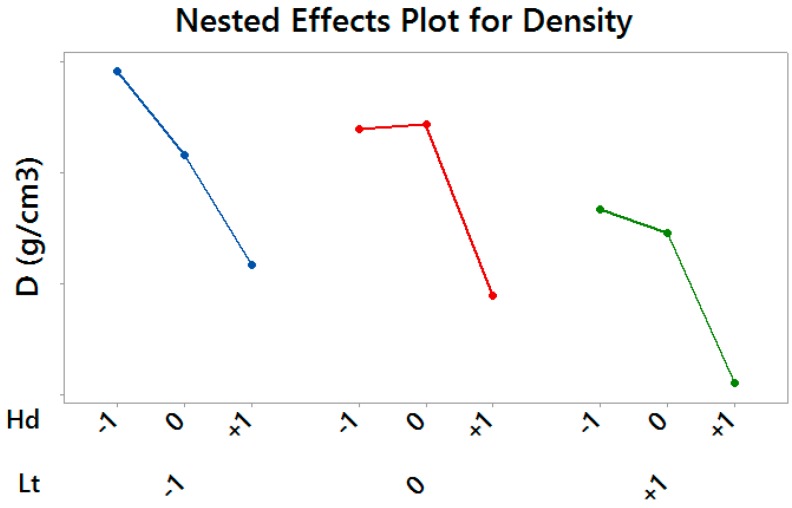
Nested Effects Plot for Density—vertical scale unit 0.01 g/cm^3^.

With regards to material density, the layer thickness and the hatch distance resulted statistically significant, with a predominant effect of the latter (*p*-value = 0.005). In particular, higher values of the hatch distance resulted in a less dense material, regardless of selected levels of the scan speed (*p*-value >> 0.05) and the layer thickness, as it can be seen looking at Figure 6, which shows the Nested Effects Plot for significant effects.

**Figure 7:**
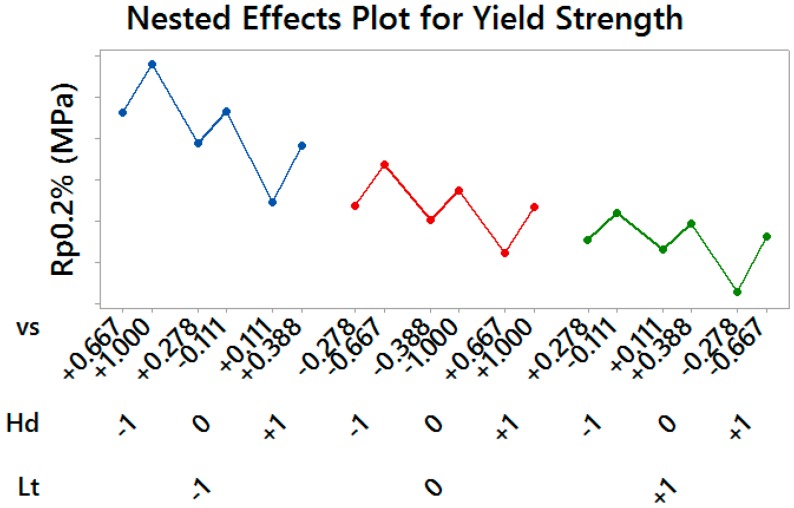
Nested Effects Plot for Yield Strength—vertical scale unit 10 MPa.

**Figure 8:**
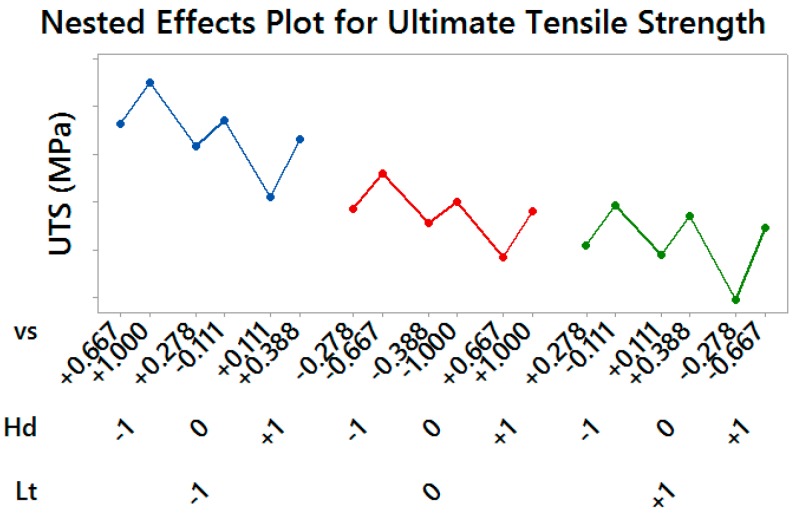
Nested Effects Plot for Ultimate Tensile Strength—vertical scale unit 10 MPa.

Material Yield Strength and Ultimate Tensile Strength showed a similar behavior, by resulting significantly affected by all considered control factors (*p*-values = 0.000): lower values of both the layer thickness and the hatch spacing produced more performing material, as well as higher values of the scan speed within each pair of treatments having the same thickness and spacing (see Figure 7 and Figure 8).

**Figure 9:**
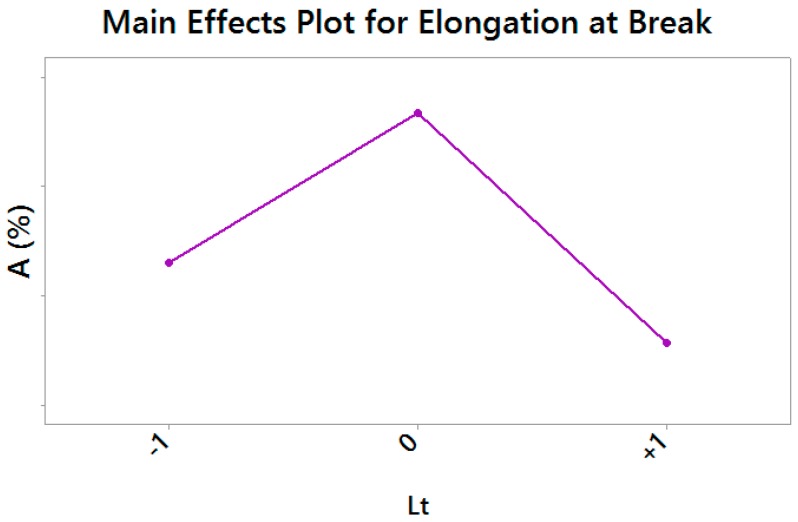
Main Effects Plot for Elongation at Break—vertical scale unit 1%.

Elongation at Break resulted the less sensitive response variable, with a strongly significant effect of the layer thickness only (*p*-value = 0.000), whose medium level returned the most ductile material, as shown by the Main Effects Plot for the only significant effect in Figure 9.

**Figure 10:**
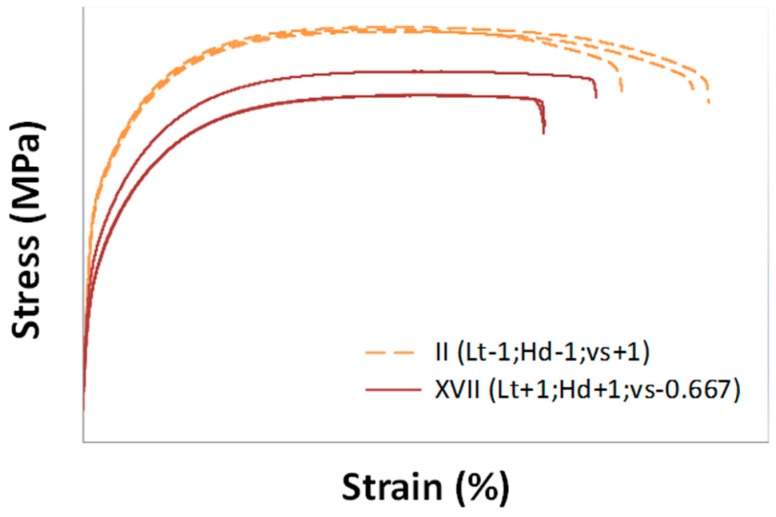
Stress–strain curves for specimens produced by using process parameters corresponding to treatments II and XVII.

Figure 10 shows stress–strain curves for specimens corresponding to treatment II, which showed the best results in terms of density and Yield Strength, and treatment XVII, which showed, on the contrary, the worst ones.

**Figure 11:**
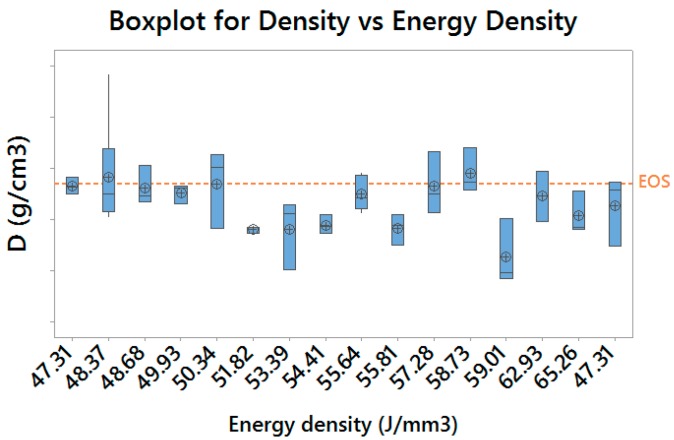
Boxplot for achieved material density (grouped by energy density)—vertical scale unit 0.02 g/cm^3^.

The results obtained are consistent with the expected ones: as long as the proper energy density is delivered to the material, it is possible to achieve an acceptable material densification, as shown in Figure 11, in which the results achieved with EOS default exposure parameters are highlighted.

### 3.2. Strategies for Process Improvement

Results obtained pointed out the possibility of tweaking laser exposure parameters to produce more performing parts having higher productivity than the reference laser exposure profile.

As expected, the use of smaller layer thicknesses and hatch distances leads to a higher density and mechanical properties. The negative effect in terms of productivity can be balanced by using higher scan speeds, which also result in tighter and cleaner “welding beads” within the sintered material.

**Figure 12:**
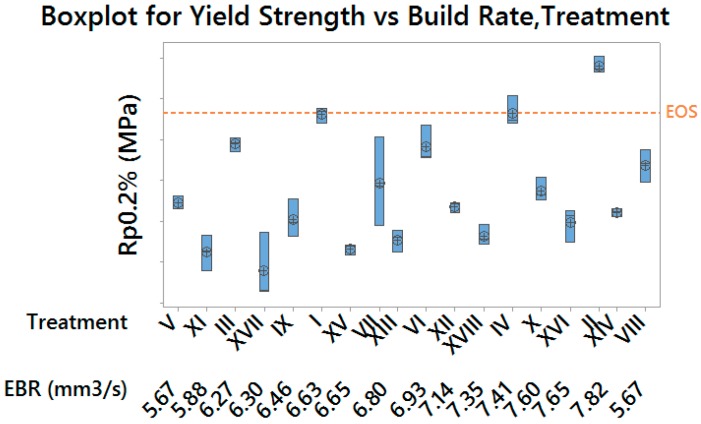
Boxplot for Yield Strength (grouped by build rate and treatment)—vertical scale unit 10 MPa.

**Figure 13:**
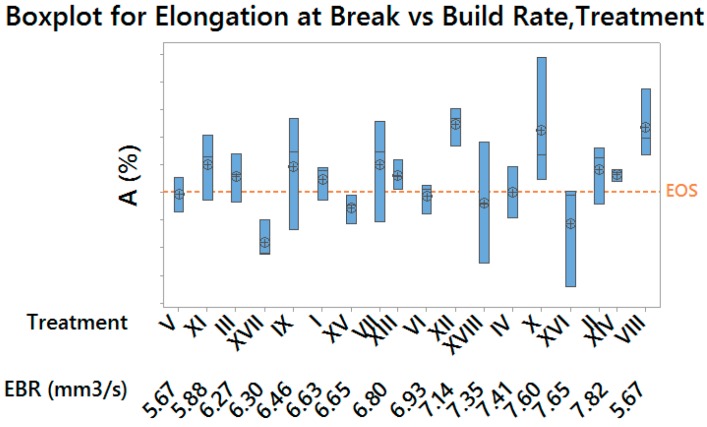
Boxplot for Elongation at Break (grouped by build rate and treatment)—vertical scale unit 1%.

Figure 12 and Figure 13 allow to compare results for the Yield Strength and the Elongation at Break (which are the most representative for material performances) in terms of process productivity. Moreover, it is possible to evaluate the variability corresponding to each experimental condition, which resulted merely physiological, as confirmed by Bartlett and Levene (for normal distribution and not normal distribution, respectively) tests for variance homogeneity. As well as for previous plots, results obtained by using default exposure profiles are highlighted.

In particular, two optimal laser parameters settings were identified, with higher build rates than the default exposure profile:

- Treatment II, which returned both higher Yield Strength and Elongation at Break, with a time saving of 3.3%, therefore suitable for a high performance production;
- Treatment VIII, which returned lower Yield Strength and higher Elongation at Break, with a time saving of 5.5%, therefore suitable for a medium performance production/prototyping.

**Figure 14:**
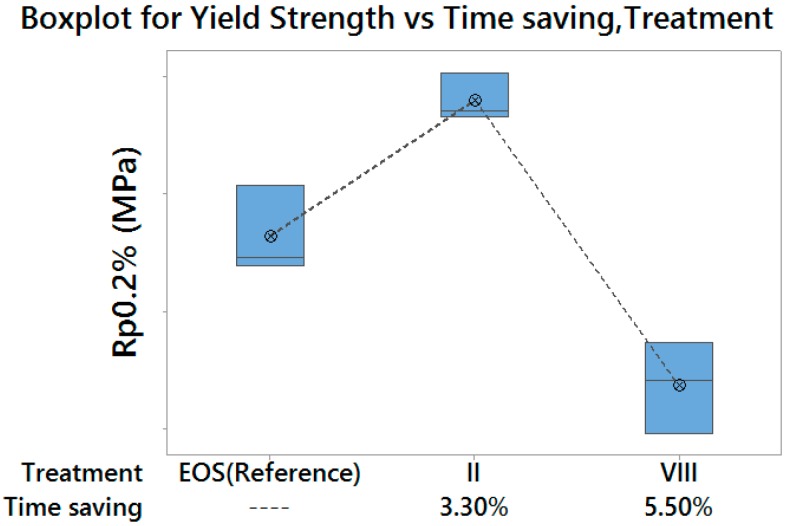
Enhancement achieved for Yield Strength—vertical scale unit 10 MPa.

**Figure 15:**
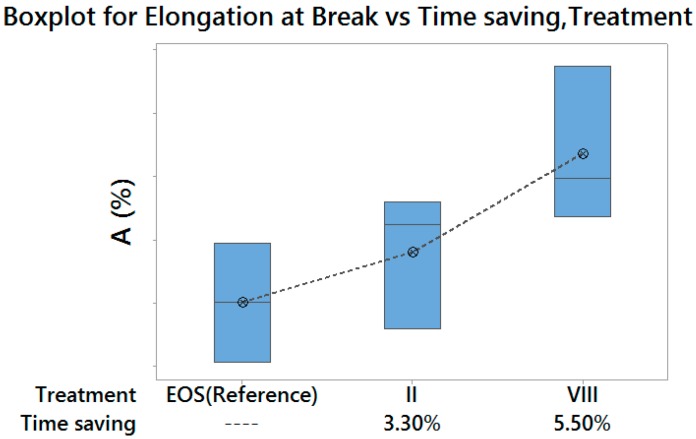
Enhancement achieved for Elongation at Break—vertical scale unit 1%.

**Figure 16:**
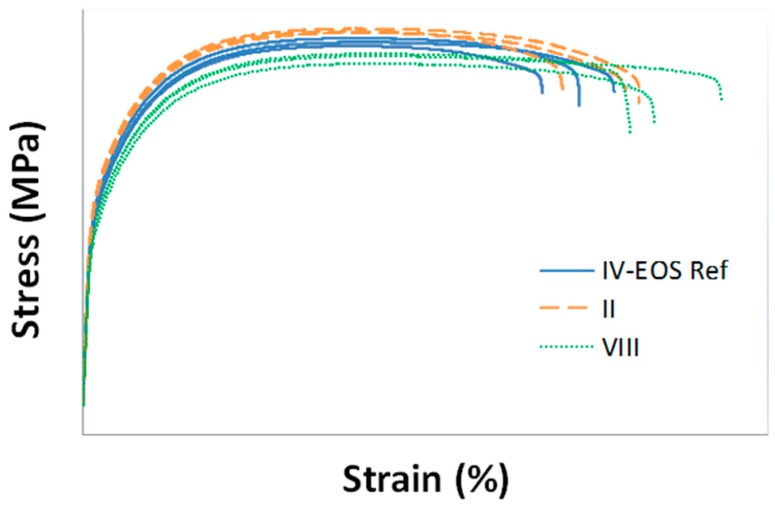
Stress–strain curves for specimens produced by using process parameters corresponding to treatments IV (default), II and VIII.

Figure 14 and Figure 15 summarize the achievable results, in terms of Yield Strength and Elongation at Break, for the two above-mentioned optimal treatments, compared with those obtained with default exposure parameters, as well as with the corresponding time saving for part production. Moreover, Figure 16 shows stress–strain curves for specimens corresponding to the three above-mentioned experimental treatments.

Despite the small loss in terms of material yield strength corresponding to Treatment VIII, it is important to highlight that the actual time saving for this treatment is even larger than 5.5%, since it is referred only to the melting time. In fact, treatment VIII is characterized by a larger layer thickness (if compared to treatments II and IV-Default), resulting in a lower number of powder layers to be spread, thus in a reduction of the time needed for re-coating operations.

## 4. Conclusions

In this paper, effects of different laser exposure strategies on the achievable tensile properties of AlSi10Mg parts produced by the DMLS technology were assessed by means of a statistical approach.

Nested ANOVA results pointed out the possibility of tweaking laser exposure parameters as long as the proper energy density is delivered to the material.

The use of smaller layer thicknesses and hatch distances led to higher density and mechanical properties. The negative effect in terms of productivity was balanced by using higher scan speeds, which also resulted in tighter and cleaner “welding beads” within the sintered material. High levels of layer thickness did not allow acceptable material consolidation and mechanical properties without productivity decay, given the available laser power.

The results obtained are consistent with those which have been found in the literature in terms of achievable consolidation of the processed material with respect to the used energy density range. The resulting mechanical properties were higher or at least comparable to those of previous experimental investigations.

In particular, two optimal laser parameters settings, with a good resulting material performance (even better than the reference exposure profile) and higher build rates, were identified. Thanks to the use of a 400 W laser source, the build rates achieved are larger than those usually found in literature, which were obtained with lower power sources (100–250 W).

With regards to the experimental approach used, it is possible to conclude that the NEM approach allows maximizing the explorable experimental region, if compared to conventional factorial methods, which would imply the use of narrow intervals in which to vary the control factors, or several experimental treatments out of the optimal process window. Moreover, data analysis through Nested ANOVA provides more insight into the effects of related factors and their significance than other techniques, such as two-way ANOVA or RSM, which can suffer collinearity issues in nested effects estimation, leading to unreliable regression models.

The above-defined experimental and statistical analysis protocols are adaptable, with appropriate adjustments, to other PBF technologies, such as the Electron Beam Melting (EBM) or the Selective Laser Melting (SLM), and other materials.

## Figures and Tables

**Table 1:** Chemical composition of EOS AlSi10Mg according to EOS datasheet (percentage in weight).

Chemical Element	Wt-%
Al	Balance
Fe	≤0.55
Si	9.0–11.0
Cu	≤0.05
Mn	≤0.45
Ni	≤0.05
Mg	0.2–0.45
Zn	≤0.10
Pb	≤0.05
Sn	≤0.05
Ti	≤0.15

**Table 2:** Part Property Profile and Stress Relieving Cycle procedure for EOS AlSi10Mg.

PPP	SRC Procedure
EOS AlSi10Mg Speed 30 μm	2 h at 300 °C—Air

**Table 3:** Nested experimental plan defined for investigation.

Treatment	L_t_	H_d_	v_s_	ψ_d_ (J/mm^3^)	EBR (mm^3^/s)
I	−1	−1	+0.667	55.81	6.63
II	−1	−1	+1	48.37	7.65
III	−1	0	+0.278	59.01	6.27
IV	−1	0	+0.667	49.93	7.41
V	−1	+1	−0.111	65.26	5.67
VI	−1	+1	+0.278	53.39	6.93
VII	0	−1	+0.111	54.41	6.8
VIII	0	−1	+0.388	47.31	7.82
IX	0	0	−0.278	57.28	6.46
X	0	0	+0.111	48.68	7.6
XI	0	+1	−0.667	62.93	5.88
XII	0	+1	−0.278	51.82	7.14
XIII	+1	−1	−0.388	54.41	6.8
XIV	+1	−1	−0.111	48.37	7.65
XV	+1	0	−0.667	55.64	6.65
XVI	+1	0	−0.388	48.68	7.6
XVII	+1	+1	−1	58.73	6.3
XVIII	+1	+1	−0.667	50.34	7.35

**Table 4:** ANOVA table of performed Nested Design.

Source of Variation	Sum of Squares	Degrees of Freedom	Mean Square
**L_t_**	$\mathrm{bcn}\overset{a}{\underset{i=1}{\sum }}{\left({\bar{y}}_{i•••}-{\bar{y}}_{••••}\right)}^{2}$	a−1	${\mathrm{MS}}_{L_{t}}$
**H_d_(L_t_)**	$\mathrm{cn}\overset{a}{\underset{i=1}{\sum }}\overset{b}{\underset{j=1}{\sum }}{\left({\bar{y}}_{\mathrm{ij}••}-{\bar{y}}_{i•••}\right)}^{2}$	a(b−1)	${\mathrm{MS}}_{H_{d}\left(L_{t}\right)}$
**v_s_(H_d_)**	$n\overset{a}{\underset{i=1}{\sum }}\overset{b}{\underset{j=1}{\sum }}\overset{c}{\underset{k=1}{\sum }}{\left({\bar{y}}_{\mathrm{ijk}•}-{\bar{y}}_{\mathrm{ij}••}\right)}^{2}$	ab(c−1)	${\mathrm{MS}}_{v_{s}\left(H_{d}\right)}$
**Error**	$\overset{a}{\underset{i=1}{\sum }}\overset{b}{\underset{j=1}{\sum }}\overset{c}{\underset{k=1}{\sum }}\overset{n}{\underset{l=1}{\sum }}{\left(y_{\mathrm{ijkl}}-{\bar{y}}_{\mathrm{ijk}•}\right)}^{2}$	abc(n−1)	${\mathrm{MS}}_{E}$
**Total**	$\overset{a}{\underset{i=1}{\sum }}\overset{b}{\underset{j=1}{\sum }}\overset{c}{\underset{k=1}{\sum }}\overset{n}{\underset{l=1}{\sum }}{\left(y_{\mathrm{ijkl}}-{\bar{y}}_{••••}\right)}^{2}$	abcn−1	

**Table 5:** Nested ANOVA results for Density (α = 0.05).

Source	DF	Seq SS	Adj SS	Adj MS	*f*-Value	*p*-Value	VIF	SI
**L_t_**	2	0.00104	0.00104	0.00052	4.31	**0.021**	1.33	0.114
**H_d_(L_t_)**	6	0.00272	0.00272	0.00045	3.75	**0.005**	1.33	0.298
**V_s_(H_d_)**	9	0.00102	0.00102	0.00011	0.94	**0.505**	1.00	0.112
**Error**	36	0.00436	0.00436	0.00012				0.476
**Total**	53	0.00916						
					**R^2^ = 52.34% − Adj R^2^ = 29.84%**			

**Table 6:** Nested ANOVA results for Yield Strength (α = 0.05).

Source	DF	Seq SS	Adj SS	Adj MS	*f*-Value	*p*-Value	VIF	SI
**L_t_**	2	6803.7	6396.4	3198.20	311.28	**0.000**	1.36	0.683
**H_d_(L_t_)**	6	1573.5	1694.2	282.37	27.48	**0.000**	1.27–1.42	0.158
**V_s_(H_d_)**	9	1252.0	1252.0	139.12	13.54	**0.000**	1.00–1.04	0.125
**Error**	33	339.1	339.1	10.27				0.034
**Total**	50	9968.4						
					**R^2^ = 96.60% − Adj R^2^ = 94.85%**			

**Table 7:** Nested ANOVA results for Ultimate Tensile Strength (α = 0.05).

Source	DF	Seq SS	Adj SS	Adj MS	*f*-Value	*p*-Value	VIF	SI
**L_t_**	2	4647.8	4425.8	2212.92	305.99	**0.000**	1.36	0.690
**H_d_(L_t_)**	6	801.1	801.1	149.28	20.64	**0.000**	1.27–1.42	0.119
**V_s_(H_d_)**	9	1052.7	1052.7	116.96	16.17	**0.000**	1.00–1.04	0.156
**Error**	33	238.7	238.7	7.23				0.035
**Total**	50	6740.3						
					**R^2^ = 96.46% − Adj R^2^ = 94.64%**			

**Table 8:** Nested ANOVA results for Elongation at Break (α = 0.05).

Source	DF	Seq SS	Adj SS	Adj MS	*f*-Value	*p*-Value	VIF	SI
**L_t_**	2	41.38	41.52	20.758	11.91	**0.000**	1.35–1.47	0.325
**H_d_(L_t_)**	6	12.16	12.11	2.018	1.16	0.351	1.27–1.33	0.095
**V_s_(H_d_)**	9	12.83	12.83	1.426	0.82	0.604	1.00–1.04	0.101
**Error**	35	61.00	61.00	1.743				0.479
**Total**	52	127.37						
					**R^2^ = 52.11% − Adj R^2^ = 28.85%**			

**Table 9:** Summary of significant effects (α = 0.05).

Control Factor	Density	Yield Strength	Ultimate Tensile Strength	Elongation at Break
**Layer Thickness, L_t_**	✔	✔	✔	✔
	*p*-value = 0.021	*p*-value = 0.000	*p*-value = 0.000	*p*-value = 0.000
**Hatch Distance, H_d_**	✔	✔	✔	✖
	*p*-value = 0.005	*p*-value = 0.000	*p*-value = 0.000	*p*-value = 0.351
**Scan Speed, v_s_**	✖	✔	✔	✖
	*p*-value = 0.505	*p*-value = 0.000	*p*-value = 0.000	*p*-value = 0.604
